# Deep Learning Algorithm Classifies Heartbeat Events Based on Electrocardiogram Signals

**DOI:** 10.3389/fphys.2020.569050

**Published:** 2020-10-02

**Authors:** Yongbo Liang, Shimin Yin, Qunfeng Tang, Zhenyu Zheng, Mohamed Elgendi, Zhencheng Chen

**Affiliations:** ^1^School of Life and Environmental Sciences, Guilin University of Electronic Technology, Guilin, China; ^2^School of Electronic Engineering and Automation, Guilin University of Electronic Technology, Guilin, China; ^3^School of Electrical and Computer Engineering, University of British Columbia, Vancouver, BC, Canada; ^4^British Columbia Children’s and Women’s Hospital, Vancouver, BC, Canada

**Keywords:** digital health, digital medicine, data science, arrhythmia detection, BiLSTM neural network, CNN – convolutional neural network

## Abstract

Cardiovascular diseases (CVDs) have become the number 1 threat to human health. Their numerous complications mean that many countries remain unable to prevent the rapid growth of such diseases, although significant health resources have been invested toward their prevention and management. Electrocardiogram (ECG) is the most important non-invasive physiological signal for CVD screening and diagnosis. For exploring the heartbeat event classification model using single- or multiple-lead ECG signals, we proposed a novel deep learning algorithm and conducted a systemic comparison based on the different methods and databases. This new approach aims to improve accuracy and reduce training time by combining the convolutional neural network (CNN) with the bidirectional long short-term memory (BiLSTM). To our knowledge, this approach has not been investigated to date. In this study, Database I with single-lead ECG and Database II with 12-lead ECG were used to explore a practical and viable heartbeat event classification model. An evolutionary neural system approach (Method I) and a deep learning approach (Method II) that combines CNN with BiLSTM network were compared and evaluated in processing heartbeat event classification. Overall, Method I achieved slightly better performance than Method II. However, Method I took, on average, 28.3 h to train the model, whereas Method II needed only 1 h. Method II achieved an accuracy of 80, 82.6, and 85% compared with the China Physiological Signal Challenge 2018, PhysioNet Challenge 2017, and Massachusetts Institute of Technology-Beth Israel Hospital (MIT-BIH) Arrhythmia datasets, respectively. These results are impressive compared with the performance of state-of-the-art algorithms used for the same purpose.

## Introduction

The heartbeat is a basic physiological phenomenon of the human body, and it is the most direct manifestation of heart function. Under the influence of age and lifestyle habits, the heartbeat may show a variety of abnormal states, such as tachycardia, bundle branch or atrioventricular blockage, and premature atrial or ventricular contraction (Roth et al., 2015; Benjamin et al., 2017). Cardiovascular diseases (CVDs) can be detected and diagnosed by electrocardiogram (ECG), a non-invasive electrophysical measurement of cardiac activity that reflects the working state of the heart in real time. In general, an ECG is obtained through a Holter monitor and a standard 12-lead setup consisting of three limb leads, three pressurized limb leads, and six thoracic leads. A complete heartbeat process is initiated by the sinus node, consisting of the depolarization of atriums and ventricles and the repolarization of ventricles, in which atrial depolarization forms a P wave, ventricular depolarization forms a QRS complex wave, and the repolarization of ventricles forms a T wave (Klabunde, 2012).

In 2019, telemedicine and mobile medicine started to be developed rapidly, resulting in their popular use and drawing attention to the auxiliary diagnosis of cardiac disease using ECG signals (Attia et al., 2019). A number of previous studies on this auxiliary diagnosis have focused on the preprocessing of ECG signals (Elgendi et al., 2017), feature extraction and analysis (Qin et al., 2017; Zhong et al., 2018), and complex classification models (Celin and Vasanth, 2018; Diker et al., 2019; Mondéjar-Guerra et al., 2019; Zhang et al., 2019). Generally, raw ECG signals contain baseline drift, power line interference, motion artifacts, muscle, and other noises. These noises affect the morphological feature recognition of ECG and can produce misdiagnosis. Many researchers use wavelet denoising, smoothing, bandpass, or adaptive filters, and other noise-filtering methods to address these issues (Luz et al., 2016; Peng and Wang, 2017).

Fundamentally, the removal of noise is the primary task of ECG signal processing to enable an accurate diagnosis. The extraction and analysis of ECG features have been widely studied with morphological features, statistical analysis of heart rate variability (HRV), time-frequency domain feature analysis, and wavelet analysis. For example, Hao et al. (2019) define many time-frequency domain parameters based on the morphological characteristics of ECG and use them to automatically recognize atrial fibrillation (AF), while Jiménez-Serrano et al. (2017) utilize particular HRV parameters to classify different heartbeat categories. Elsewhere, Pławiak (2018) conducts a fast Fourier transform and spectral density analysis of ECG signals and identifies the frequency domain features that contain very low, low-, or high-frequency power to classify heartbeats in AF.

With the development of machine and deep learning technologies, more ECG category classification models have been proposed, enabling the automatic diagnosis of different heart diseases. Of these technologies, the support vector machine (SVM) approach (Li et al., 2019), random forest method (Yang et al., 2020), autoregressive modeling (Ge et al., 2002), artificial (Xu et al., 2015), and convolutional neural networks (CNNs; Kiranyaz et al., 2016; Hannun et al., 2019), and long short-term memory (LSTM) have been mainly used to establish ECG classification models (de Chazal et al., 2004; Asl et al., 2008; Acharya et al., 2017; Yildirim, 2018). To date, several heartbeat abnormalities have been frequently studied, such as AF, premature ventricular contraction (PVC), paced beat, and left or right bundle branch block (LBBB or RBBB, respectively; Raj and Ray, 2018). However, many studies have focused on two or three ECG categories and different heart disease classifications, making them difficult to compare and limiting the application of the resulting diagnosis models. In addition, with the development of mobile medical technology, a large number of wearable device data are obtained. In view of the demand for wearable health devices for rapid detection and evaluation, researchers cannot simply pursue high performance and ignore the problem of computational complexity. An excellent algorithm model should achieve a balance between performance and computational complexity. Therefore, a calculation challenge is proposed for fast and accurate disease detection.

To explore and achieve the classification of multiple heart diseases, a deep learning model for automatic diagnosis was constructed and tested on three different datasets. An evolutionary neural system approach was also developed following Pławiak’s (2018) paper for comparison with the proposed deep learning approach.

## Materials and Methods

### Database I

Two independent databases were used in this study. Database I is an ECG segment dataset collected by Pławiak (2018) from the MIT-BIH Arrhythmia Database containing 1,000 10-s single-lead ECG segments (Moody and Mark, 2001). Each ECG segment is uniquely medically classified across 17 types: normal sinus rhythm, pacemaker rhythm, and 15 categories of cardiac dysfunction.

### Database II

Database II is an ECG segment dataset with 12-lead ECG signals shared by the China Physiological Signal Challenge 2018 (Liu et al., 2018). Database II is also included in the PhysioNet Challenge 2020 database (Perez Alday et al., 2020). This dataset comprises 6,877 12-lead ECG recordings that can be utilized for the automatic identification of rhythm abnormalities, with 53.7% taken from male and 46.3% from female patients. Database II contains one normal and eight abnormal heartbeat categories: (1) normal, (2) AF, (3) first-degree atrioventricular block (I-AVB), (4) advanced LBBB (LBBB), (5) advanced RBBB (RBBB), (6) premature atrial contraction (PAC), (7) PVC, (8) ST-segment depression (STD), and (9) ST-segment elevation (STE). The length of the ECG signal ranges from 10 to 60 s, and the sampling rate is 500 Hz.

### Machine and Deep Learning Approaches

#### Method I

In this study, a recently published evolutionary neural system approach (Method I) that combines a genetic algorithm (GA) and the machine learning method proposed by Pławiak (2018) was used as a benchmark to recognize heartbeat categories. The evolutionary neural system extracts power spectral density features using a Hamming window with 512 samples width and Welch’s method. Then, a GA and SVM were used to optimize the gamma (-g) and nu (-n) parameters of the SVM classifier based on power spectral density features. After the optimization process, the optimal gamma and nu values were acquired. Finally, the evolutionary neural system with optimal parameters was achieved.

#### Method II

This study proposed a deep learning approach based on a sequential ECG signal with rich waveform morphology to recognize heartbeat categories. Using several CNN blocks to extract the convolutional features of the ECG signal and bidirectional long short-term memory (BiLSTM) to determine the convolutional features, the deep learning approach proposed in this study is a combination of CNN and BiLSTM. More detailed processes and implementation methods are presented below.

#### Preprocessing

In the preprocessing stage, there was no need to prefilter the signals in Method I or Method II, but a scaling process was required to make the signal range from -1 to 1 through the min–max normalization method.

#### Segmentation

**FIGURE 1:**
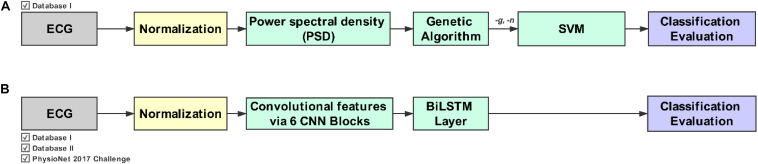
Workflow for the evolutionary neural system approach (Method I) and the deep learning approach (Method II). **(A)** Method I combines the genetic algorithm (GA) with the support vector machine (SVM). **(B)** Method II combines the convolution neural network (CNN) with the bidirectional long short-term memory (BiLSTM) network. Note that Method I requires a feature extraction phase in addition to the GA and SVM.

Database I had the same length, which was 10 s, so it did not need the segmentation process. However, the recordings in Database II had different lengths, ranging from 10 to 60 s. To achieve consistency with ECG signal processing, a fixed length of ECG segments was necessary in the model. A 30-s length was used in Database II. For the recordings with more than 30 s, the signals for the first 30 s were retained, and the rear signal was discarded. For the recordings with less than 30 s, zero padding technology was used, and zeros were introduced before the signal as padding. A workflow of the evolutionary neural system and deep learning approach is presented in Figure 1.

#### Evolutionary Neural System Approach

Solving problems through a GA is similar to biological evolution. Replication, mutation, and other operations are used to produce the next generation, gradually eliminating the solution of low fitness function value and increasing the solution of high fitness function value. After the evolution of N generation, acquiring individuals with high fitness function values is possible. A GA is widely used in many optimization applications. Machine learning plays an important role in many fields of data analysis and has a good effect on classification, clustering, regression, and other issues (Mincholé and Rodriguez, 2019).

The SVM classifier (Celin and Vasanth, 2018) is a highly powerful tool for dealing with classification issues. It aims to minimize structural risk using the concept of margin, so its decision boundary is the maximum margin hyperplane for the learning sample solution. However, determining the hyperplane for non-linear classification is not possible. Although SVM extends the applicability of a linear classifier to non-linear separable data by using the kernel method, it is basically used as a binary classifier. Generally, SVM realizes multi-category classification by using one-versus-rest and one-versus-one extensions.

For the evolutionary neural system approach, Pławiak (2018) combined a GA and SVM (Pławiak, 2018). Through repeated optimizing, training, and testing, the evolutionary neural system approach can acquire the optimal parameters for fitness function.

Power spectral density features were extracted using the aforementioned approach. A Hamming window with 512 samples and Welch’s method were applied. For a single segment of ECG signal, a feature vector with a length of 4,001 frequency components was obtained.

Method I was constructed based on a GA and SVM. The core of Method I was parameter optimization. Gamma (-g) and nu (-n) were the SVM parameters that needed to be optimized. The optimization process was as follows: (1) Gamma and nu parameters for initial generation were randomly produced. (2) The classification error of SVM was set as the fitness function, and the target value of the fitness function was 0. (3) The generation number and the number of individuals in the population were set; the maximum number of generations was 30, and the number of individuals in the population was 50. (4) The dataset was processed repeatedly by crossover (intermediate type), mutation (uniform type), and selection (tournament method). The probability of crossover was 0.7, and the probability of mutation was 0.3. (5) The optimal gamma (2.52e-5) and nu (0.0207) parameters were obtained with minimal classification error.

#### Deep Learning Approach

The CNN approach is rapidly growing in popularity and is widely used in image, text, audio, and video processing. Similar to other neural nets, CNNs consist of input and output layers along with multiple hidden layers in between. These layers focus on learning data features, the three most common being the convolutional, activation, and pooling layers. The layers contain a series of convolutional filters that can activate and learn some features of the input data. Through repeated extraction from dozens of convolutional layers, each layer learns the different features. Rather than extracting morphological features (e.g., amplitude, peaks, durations), a CNN can automatically extract rich features, providing apparent advantages.

Recurrent neural networks (RNNs) are an effective deep learning approach for processing time-series signals. An RNN differs from other kinds of neural nets in that it establishes a weight connection in the neurons of its hidden layers. As the sequence advances, the information of one hidden layer is transferred to the next. An RNN therefore shows excellent performance compared with traditional neural networks in predicting new sequences based on historical sequence data. However, although the RNN establishes a weight connection between the hidden layers’ neurons, weight transmission has a short-term memory (Zhu et al., 2019). On this basis, an LSTM net combines short- and long-term memory by introducing gate control, which solves gradient disappearance to a certain extent. The core units of LSTM are cell states that include input, forget, and output gates (Reddy and Delen, 2018). Because long-term memory is improved, an LSTM network can understand long-term historical information better and apply it to new predictions, thereby optimizing the deficiencies of the standard RNN structure.

Although an LSTM network can remember and understand historical data, it does not effectively use new data to help with final predictions. As such, further adjustment is required to enable the network to have both forward and reverse computing capabilities, resulting in a two-way LSTM network. A BiLSTM (Liu and Guo, 2019) with two-way capabilities was therefore constructed based on the LSTM network. BiLSTM networks have shown excellent performance in sequence prediction modeling compared with different RNN and LSTM structures, especially in the fields of machine translation and speech or handwriting recognition (Rao et al., 2018). For this study, heartbeat categories were identified from the 12-lead ECG data, and there were intrinsic relationships between the signals from different leads. A BiLSTM approach can be used as an intermediate step between the CNN features and the classification layer to improve accuracy by selecting the optimal features from the CNN.

**FIGURE 2:**
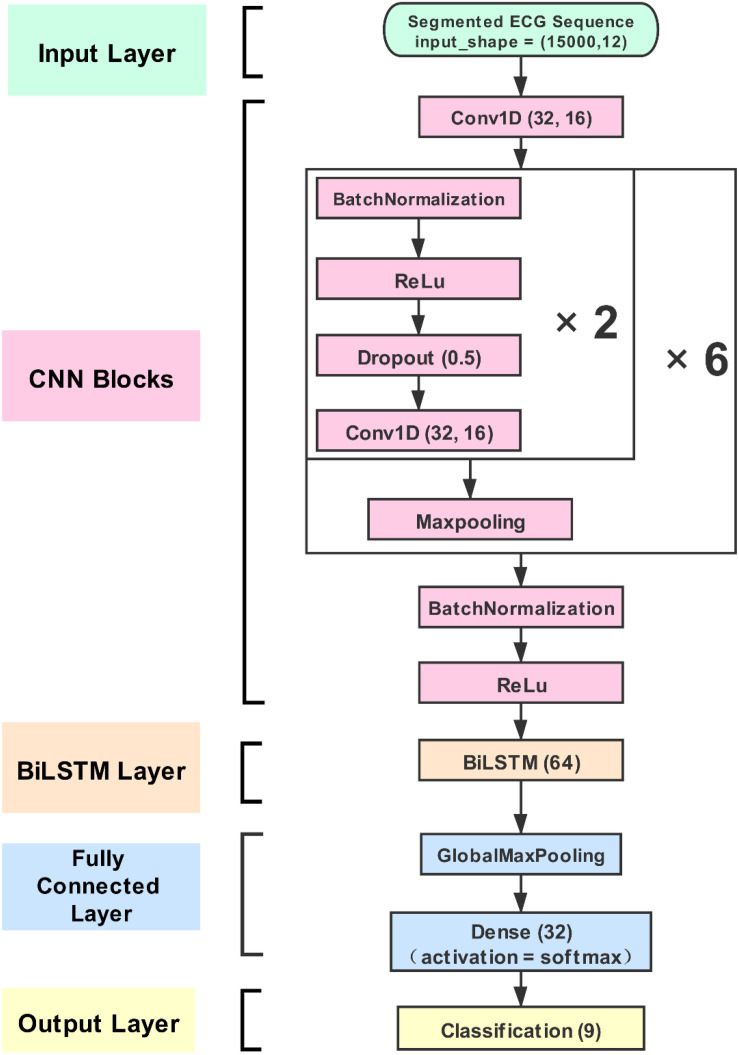
The proposed convolution neural network (CNN)-bidirectional long short-term memory (BiLSTM) structure. The CNN strategy can extract more convolution features from electrocardiogram (ECG) signal, and the BiLSTM strategy can learn effectively by selecting from the optimal features extracted to improve classification accuracy. The proposed model is generic and can be easily used for other applications.

A CNN-BiLSTM network was constructed for this study. This approach consists of four layers: (1) the input layer, (2) the CNN blocks, (3) the BiLSTM layer, and (4) the classification layer. The segmented ECG time-series signals (12 channels) and 15,000 samples were fed into the input layer. The signals were calculated with a one-dimensional convolution and then outputted to the CNN calculation blocks. The blocks automatically extracted the signals’ deeper features and constructed a feature matrix. In the blocks, the filters and kernel sizes are 32 and 16, respectively. The padding type is “same.” The dropout layer is also set in the blocks, and the dropout rate is 0.5. This was an effective means to solve the overfitting problem (Srivastava et al., 2014). As mentioned earlier, BiLSTM can be more effective in discerning feature difference and recognizing categories for the feature matrix of the ECG signal. BiLSTM contains 64 memory units. In the last stage, the densely connected layers achieve the categories’ output. The detailed framework of the CNN-BiLSTM net is shown in Figure 2.

A 10-fold cross-validation method and the random oversampling (ROS) method were used to effectively and objectively evaluate all models in training and testing (Figure 1). The dataset was first divided into 10 parts according to their categories (nine parts trained and one part tested). In other words, each class was divided into 10 parts independently from the minority to the majority class, which can ensure that the train set and the test set include samples of each category. In the training stage, the train set was processed with the ROS method. After 10 iterations, each part was tested as a test set, and the final performance was calculated across all sets. The F1 score, Sensitivity, and Specificity were used as performance evaluation measures, which are calculated as follows:

$$F\,1=\frac{\text{TP}}{\text{TP}+0.5\,\left(\text{FP}+\text{TP}\right)},$$

$$\;\,\text{Sensitivity}=\frac{\text{TP}}{\text{TP}+\text{FN}},$$

$$\;\,\text{Specificity}=\frac{\text{TN}}{\text{TN}+\text{FP}},$$

where TP stands for true positives, FP stands for false positives, TN stands for true negatives, and FN stands for false negatives.

The evolutionary neural system approach was implemented using MATLAB R2018b software installed on a Windows 10 platform. The CNN-BiLSTM net was implemented using Python 3.6 and Keras 2.2.4, which itself uses a TensorFlow 1.14 backend. For consistency, all algorithms were tested and evaluated on the same computer (Intel Core i9-9900K CPU with 3.6 GHz main frequency and 64 GB memory). MATLAB contains the Signal Processing, Wavelet, Statistics and Machine Learning, and Deep Learning toolboxes.

Note that the three datasets used in this study were different in terms of the number of ECG lead and number of categories; therefore, each dataset needed a separate training phase. Meanwhile, the cross-validation processing and the structure of the deep learning model are the same. However, the input layer and the output layer required adjustment. For example, the number of input channels for Database II was set to 12 because it is a 12-lead ECG database. In contrast, the number of input channels for Database I and PhysioNet Challenge 2017 database was set to 1 because these databases contain single-lead ECG signals. Besides, the number of output categories for Database I, Database II, and PhysioNet Challenge 2017 database was 17, 9, and 4, respectively.

## Results

In this study, a comparison between Method I and Method II was conducted first to process Database I. In Database I, there are 17 heartbeat categories, and the dataset is unbalanced. The normal sinus rhythm had 283 ECG segments, but several categories had only 10 ECG segments. Method I and Method II were applied to Database I. Table 1 shows the classification performance of both methods.

**TABLE 1:** Classification performance of Method I and Method II on Database I.

**No.**	**ECG Categories**	**#Num**	**Sensitivity (%)**		**Specificity (%)**		***F*_1_ Score (%)**	
			**Method I**	**Method II**	**Method I**	**Method II**	**Method I**	**Method II**
**1**	Normal sinus rhythm	283	91	71	89	94	90	66
**2**	Premature atrial beat	66	55	85	66	96	58	72
**3**	Atrial flutter	20	75	89	100	100	83	91
**4**	Atrial fibrillation	135	93	69	96	99	94	75
**5**	Supraventricular tachyarrhythmia	13	75	58	100	100	83	74
**6**	Pre-excitation (WPW)	21	100	100	100	100	100	100
**7**	Premature ventricular contraction	133	85	46	78	98	81	52
**8**	Ventricular bigeminy	55	82	93	75	100	78	94
**9**	Ventricular trigeminy	13	65	67	75	100	67	76
**10**	Ventricular tachycardia	10	100	100	100	100	100	95
**11**	Idioventricular rhythm	10	100	78	100	100	100	88
**12**	Ventricular flutter	10	100	100	100	100	100	95
**13**	Fusion of ventricular and normal beat	11	100	70	100	99	100	70
**14**	Left bundle branch block beat	103	95	100	100	100	97	96
**15**	Right bundle branch block beat	62	100	100	100	100	100	96
**16**	Second-degree heart block	10	100	100	100	100	100	100
**17**	Pacemaker rhythm	45	100	100	100	100	100	98
	**Mean**		**90**	**84**	**93**	**99**	**90**	**85**

*Note that the bold values refer to the overall performance of Method I and Method II.*

There is a trade-off between sensitivity and specificity for both methods. For example, Method I showed better sensitivity than Method II in detecting normal sinus rhythm, AF, supraventricular tachyarrhythmia, PVC, idioventricular rhythm, and fusion of ventricular and normal beat. Method II showed better sensitivity than Method I in detecting PAC, atrial flutter, ventricular bigeminy, ventricular trigeminy, and LBBB. Meanwhile, six categories were detected with 100% sensitivity by Method I and Method II. Generally, for arrhythmia detection, Method I was more applicable to detect AF, PVC, and idioventricular rhythm. Method II was more practical to detect PAC, atrial flutter, and ventricular bigeminy. For other arrhythmia categories, Method I and Method II had a similar or equivalent sensitivity.

On the other hand, Method I only achieved higher specificity in detecting fusion of ventricular and normal beat when compared to Method II. With regard to the general performance, although the overall *F*_1_ score of Method II is 5% lower than that of Method I, the processing time of Method II is 1.1 h vs. 37 h for Method I. Note that dealing with a large amount of data processing time becomes a crucial challenge, especially for large recorded (collected over a week or more) ECG signals.

**FIGURE 3:**
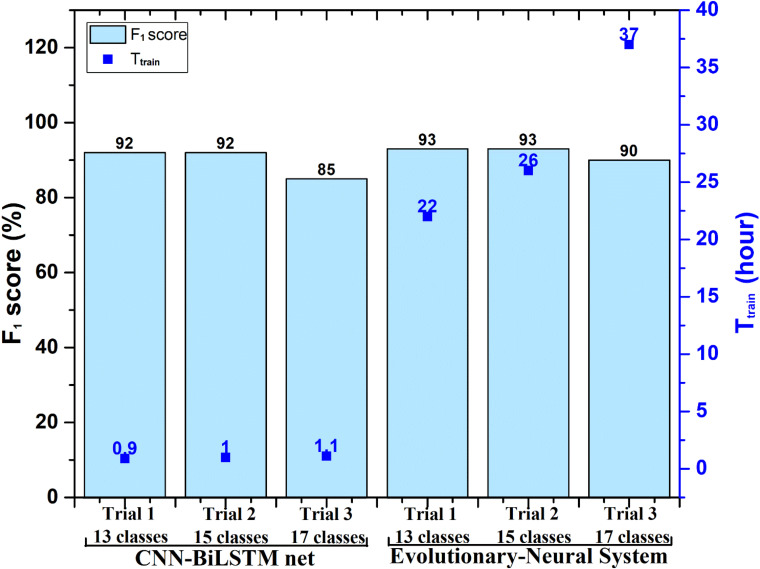
Performance comparison between Method I (evolutionary neural system approach) and the proposed Method II [convolution neural network (CNN)-bidirectional long short-term memory (BiLSTM) approach] in processing Database I. T_*train*_, training stage time of the evolutionary neural system and proposed CNN-BiLSTM. Note that the testing time (T_*test*_) of Method I and Method II is less than 10 s, which is not shown in the figure.

In Pławiak (2018) research, he found that some dysfunctional classes would affect the classification; he attempted to remove them and conduct other trials. Three classification trials were also performed in Pławiak’s (2018) research, and they contained 17, 15 (without supraventricular tachyarrhythmia and fusion of ventricular and normal beats), and 13 classes (same as 15 but without PVC and ventricular tachycardia). Here, Methods I and II were also applied in the same classification trials. All processing was done on the same computing device. Figure 3 shows the classification performance and time consumption of Methods I and II. The *F*_1_ score (*F*_1_) was used as a classification performance measure.

In Figure 3, the *F*_1_ score of Trial 1 (13 classes) and Trial 2 (15 classes) using Method I was 93%, whereas the *F*_1_ score of Trial 1 and Trial 2 using Method II was 92%. The classification performance of the two methods was very similar. However, the time consumption of Method II remained about 1 h, whereas Method I needed more than 20 h. The time consumption for Method I was very large, although Database I included only 1,000 ECG segments (a 10-s duration for each segment).

Database II includes 6,877 recordings; each recording has a 10- to 60-s 12-lead ECG signal segment. One normal and eight abnormal heartbeat categories are labeled. The original plan was to apply Method I and Method II on Database II. However, because of the large quantity of data, Method I failed to finish processing Database II. Method II successfully classified the heartbeat categories. Table 2 shows the classification performance of nine heartbeat types. The *F*_1_ score of RBBB reached 94.3%, the *F*_1_ scores for six heartbeat categories were higher than 80%, and the overall *F*_1_ score was 80%.

**TABLE 2:** Classification performance of Method II on Database II.

No.	ECG Categories	#Num	Sensitivity (%)	Specificity (%)	*F*_1_ Score (%)
**1**	Normal sinus rhythm	918	75.8	96.5	76.2
**2**	Atrial fibrillation	1,221	73.6	98.1	89.5
**3**	First-degree atrioventricular block	704	82.9	99.2	87.2
**4**	Left bundle branch block	193	65.0	99.6	83.3
**5**	Right bundle branch block	1,609	95.9	93.1	94.3
**6**	Premature atrial contraction	572	82.5	97.0	78.4
**7**	Premature ventricular contraction	649	78.8	97.6	82.1
**8**	ST-segment depression	810	75.9	97.4	81.8
**9**	ST-segment elevated	201	38.1	98.8	47.1
	**Mean**		**74.3**	**97.5**	**80.0**

*Note that the bold values refer to the overall performance of Method II.*

## Discussion

In clinical applications, doctors often need to diagnose and recognize heartbeat events based on single-lead or multiple-lead ECG signals. However, due to the heavy workload of medical diagnosis and the difference of doctors’ experience levels, an automatic heartbeat event recognition model is needed that performs efficiently with less computational complexity. One novel way to approach this would be to combine CNN with BiLSTM and to investigate how this combination performs for heartbeat events detection and classification.

Many other studies have been conducted on the classification of heartbeat categories based on ECG signals. The evolutionary neural system approach is an excellent solution to classify heartbeat categories. Based on Database I with single-lead ECG and Database II with 12-lead ECG, this study carried out a classification study using an evolutionary neural system approach (Method I) and a CNN-BiLSTM deep learning approach (Method II). Method I, proposed by Pławiak (2018), combines a GA with SVM to optimize classification performance by repeatedly optimizing classifier parameters. For classifying heartbeat categories, this study presents a deep learning network combination of CNN and BiLSTM, which differs from Method I in that it does not require repeated parameter optimization. To explore the advantages and disadvantages of Methods I and II, each method carries out the same classification using Database I, and all the processing is performed on the same computer. From Table 1, we learn that Method I achieved 5% higher classification performance than Method II, which indicates the advantage of Method I in searching for optimal results. However, Method I also showed a clear deficiency as the training phase is very time-consuming. For Database I, processing 1,000 ECG segments using Method I takes about 37 h, but Method II needs only 1.1 h to finish all the processing.

A much larger dataset would be beneficial to train and test; particularly as Database I has several categories with only 10 or 20 recordings. This type of limitation is a significant hindrance for deep learning models because larger datasets help the model learn the patterns. Accordingly, the *F*_1_ results of Trial 1 (13 classes) and Trial 2 (15 classes), in which the classes with the fewest recordings were removed, improve to 85 and 92%. As a whole, Method II achieves classification performance that is similar to Method I in Pławiak’s (2018) Trial 1 (13 classes) and Trial 2 (15 classes). The time consumption of Method II is about 1 h, but Method I still takes more than 20 h, as shown in Figure 3.

Based on this comparison, for the classification of small datasets, Method I has a distinct advantage in achieving the best classification performance. However, for the classification of large datasets, Method I has serious disadvantages. Its huge time consumption limits its application in many fields, and in some cases, it cannot even work properly. With the development of wearable devices, more and more clinical medical data need to be processed. Thus, the evolutionary neural system approach will not be the best solution for the era of big data. Instead, a solution with good performance and time consumption will be the most practical and beneficial.

The CNN-BiLSTM deep learning approach proposed in this study combines CNN with RNN. It has a deeper network depth and a considerable ability to extract and learn ECG convolutional features. In the processing of Database I, it showed accurate and acceptable classification performance and impressive time consumption. Database II is a larger clinical medical ECG dataset with 6,877 ECG recordings, with each recording showing a 12-lead ECG signal. Both Methods I and II were scheduled to process the Database II classification. However, as explained above, Method I failed to finish the classification. Method II successfully classified the heartbeat categories of Database II. As can be seen in Table 2, the overall F1 score reached 80%, and six categories scored above 0.800, with that of RBBB reaching 94.3%. Moreover, Tables 1, 2 show that our proposed deep learning model (Method II) kept a high specificity, which was 99% for Database I and 97.5% for Database II. In a wearable ECG device context where most users are not patients, Method II could play a significant role in identifying subjects who are not suffering from arrhythmias, mainly if the technology is being used daily.

There are some data imbalance problems in the dataset of this study, and many categories need to be classified. However, the overall performance of the models is stable and acceptable. The following three considerations helped us. First, we applied cross-validation technology, which made the model use the limited dataset fully. Second, we split samples as categories when the dataset was divided into the train set and test set. When the dataset is small, sampling randomly is important. Third, when we processed the Database II, we formed a validation set by sampling 50 samples randomly from each category in the training stage. A balanced validation set was benefitted to output a stable and optimal trained model.

However, we also observed that the classification of the STE category was performed poorly, and more analyses needed to be conducted. The dataset was found to be unbalanced in that the number of STE cases (201) represents just 12.5% of RBBB recordings (1,609). Given that larger quantities of data benefit the learning process for a deep learning model, more effective data balancing or augmentation should be studied and used. Second, STE is usually diagnosed by calculating information defined by medical experts and often presents much smaller morphological changes in ECG PQRST waves than AF, RBBB, or LBBB (Smith, 2001). Future work will focus on investigating morphology changes using machine learning and deep learning algorithms.

To validate the proposed deep learning model in processing the different source ECG database, the PhysioNet Challenge 2017 database (Goldberger et al., 2000; Clifford et al., 2017) is also used for comparison. The database contained 8,527 single-lead ECG segments with four categories: normal (5,154), AF (771), other rhythm (2,557), and noise (46). For this database, we used the same process and only fed the ECG time-series segment as the input of the model using the same computer. Finally, we achieved the overall F1 score of 0.826, which was the top-ranking result on the PhysioNet Challenge 2017 dataset, as shown in Table 3.

**TABLE 3:** Performance comparison between the proposed deep learning model in this study and previously tested algorithms on the PhysioNet Challenge 2017 dataset (Goldberger et al., 2000).

Rank	Year	Authors	Algorithm	*F*_1_ score (%)
**=1**	**2020**	**This study**	**CNN and BiLSTM**	**82.6**
=1	2017	Teijeiro et al. (2017)	Feature engineering and LSTM	83.1
=1	2017	Datta et al. (2017)	Feature engineering and AdaBoost	82.9
=1	2017	Zabihi et al. (2017)	Feature engineering and Random Forest	82.6
=1	2017	Hong et al. (2017)	Feature engineering and XGBoost	82.5

**The database contains 8,527 single-lead electrocardiogram (ECG) segments with four categories, which are normal sinus (5,154), atrial fibrillation (AF; 771), other rhythms (2,557), and noise (46). Note that all algorithms are ranked #1 according to the Challenge evaluation measure, as the rounding value of all F_1_ scores is 83%. The PhysioNet Challenge 2017 website provided the ranking. Note that the F_1_ score of this study was achieved by the 10-fold cross-validation method. BiLSTM, bidirectional long short-term memory; CNN, convolution neural network; LSTM, long short-term memory.* Note that the bold font highlights the performance of the proposed method (Method II).*

In recent studies, more and more researchers are trying to combine the advantages of automatic feature extraction, machine learning, CNN, RNN, and other technologies in order to build deep learning solutions for different purposes, especially in the field of time-series physiological data mining (Parvaneh et al., 2019). For ECG beat classification, traditional research mostly uses complex feature engineering methods with high computational complexity to process signal and extract features. Generally, the time consumption for feature construction and extraction is usually more than half of the whole research time. With more and more medical data waiting to be mined, improving performance with acceptable computational complexity for algorithms and models is urgent. Li et al. (2019) propose combining CNN and SVM to build an AF recognition model, which improves model performance from 93 to 96% compared with the CNN model. Swapna et al. (2018) have attempted to compare CNN net with a combination of CNN and LSTM in processing a diabetes database. The result showed that the combination of CNN and LSTM improved accuracy from 93 to 95%.

In this study, the CNN-BiLSTM deep learning approach succeeded in processing the single-lead ECG dataset and 12-lead ECG dataset, achieving proper and acceptable classification performance with a relatively lower time frame. It has obvious advantages, including the low demand for handcrafted signal processing, quick deployment of a well-trained model, and easy expansion to include more classification categories. These advantages provide greater choice in cardiovascular diagnostic methods. However, some disadvantages are equally worthy of consideration. The signal length and number of ECG leads required may not correlate with real-life examples, and a powerful computational ability and longer training time are required. Nevertheless, more and more clinical physiological data are being collected and mined, and more powerful Graphics Processing Units (GPUs) are being widely used. Although the training time of the CNN-BiLSTM deep learning approach is long, the testing phase is extremely fast compared with the benchmark algorithm.

Future research will focus on four key areas. First, a more effective algorithm to transform ECG signals and improve the validity of the model’s automatic extraction will be studied. Second, data augmentation and dataset balance will be explored. Third, greater consideration of local feature extraction (e.g., STE heartbeats) will be made. Finally, the proposed model will be used to test and validate other larger datasets.

## Conclusion

This research aimed to identify a solution for the quick and reliable classification of heartbeat categories based on ECG signals. The proposed CNN-BiLSTM deep learning model was compared with a recently published evolutionary neural system approach. The latter was found to be slightly more accurate in classifying heartbeat categories, but it was extremely slow. It took an average of 28.3 h training time over three different classification trials, whereas the deep learning approach only took, on average, 1 h. When tested using a very large database, the evolutionary neural system approach could not even complete the process. The CNN-BiLSTM model, on the other hand, was able to process the data nearly as quickly as it did for the smaller dataset, and it achieved good performance with an overall mean F1 score of 80%. Adding the BiLSTM to the extracted features from the CNN improved classification accuracy. The proposed method is a generic method that could be used for other biosignal applications.

## Data Availability Statement

Database I is publicly available via this link: https://data.mendeley.com/datasets/7dybx7wyfn/3, Database II is publicly available via this link: http://2018.icbeb.org/Challenge.html, and the PhysioNet Challenge 2017 database is publicly available via this link: https://www.physionet.org/content/challenge-2017/1.0.0/. The code can be downloaded via this link: https://github.com/Elgendi/ECG-Hearbeats-Classification-using-CNN-BiLSTM.

## Author Contributions

YL developed the algorithm and drafted the manuscript. ZC advised and supervised the project. SY conducted the data analysis and signal processing. QT and ZZ implemented the model construction. ME designed the study protocol and supervised the project and was the main contributor to the writing of the manuscript. All authors contributed to the article and approved the submitted version.

## Conflict of Interest

The authors declare that the research was conducted in the absence of any commercial or financial relationships that could be construed as a potential conflict of interest.
